# Building a Human Brain for Research

**DOI:** 10.3389/fnmol.2020.00022

**Published:** 2020-02-18

**Authors:** Maina Bitar, Guy Barry

**Affiliations:** ^1^QIMR Berghofer Medical Research Institute, Herston, QLD, Australia; ^2^The School of Biomedical Sciences, The University of Queensland, Brisbane, QLD, Australia

**Keywords:** human brain, iPSC, organoid, assembloid, microfluidics, ethics

It is vital for our understanding of human-specific development, behavior, cognition, and disease that we possess a reliable, manipulatable, and accurate experimental model of the human brain. Historically, the ability to view and manipulate a human brain model system on a molecular scale in real-time was not achievable. However, recent advances in stem cell technologies make it possible to reproduce, at least partly, human brain development in a laboratory. We are now able to replicate human neural cell types, distinct brain regions and produce organoids from embryonic and induced pluripotent stem cells. Here, we address the main developments in producing multiple neural cell types and organoids and discuss how these technologies are allowing an effective way forward to gain functional insight into human brain development and disorders. We conclude with a brief discussion on potential upcoming ethical implications of this rapidly progressing field.

## 2D *in vitro* Models of Human Brain Cells and Networks

**Figure 1:**
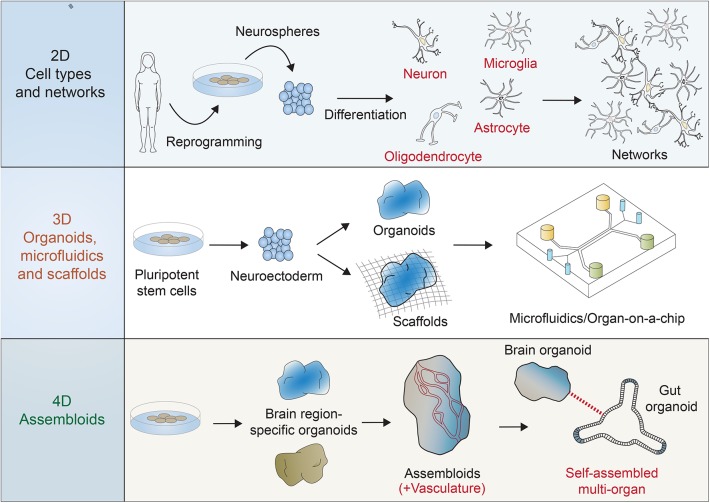
Multiple systems are possible for the use of reprogrammed human stem cells in the investigation of human brain development, function, and disease. Two dimensional (2D) functional, activatable, and manipulatable networks can incorporate many neuronal subtypes (e.g., motor neurons, cortical neurons, and interneurons) and other neural cell types (e.g., astrocytes, oligodendrocytes, and microglia). Three dimensional (3D) networks can be formed using scaffolds and microfluidic devices to include the features of 3D regionalization, whereas some organoid models can also be scaffold-free and free-floating. Four dimensional (4D) “assembloids” can expand 3D models by integrating vasculature, for example, and combining other body region organoid models, such as the gut in order to mimic the brain/gut axis.

The human brain, similar to all other species that have a brain structure, has specific gene expression patterns, pathways, and cell types that make it unique. Although there are many similarities across species, it is the differences that are needed to be considered when investigating species-specific development and disease. Until recently, research on the human brain could only be conducted using post-mortem tissue or imaging or by establishing parallels to observations made using animal models. Although useful, these techniques cannot yield vital molecular information of dynamic neural processes particular to humans. In 2006, a breakthrough in the field was achieved by Takahashi and Yamanaka, who first performed the conversion of mouse fibroblasts into induced pluripotent stem cells (iPSCs) (Takahashi and Yamanaka, 2006), a process which was soon after replicated using human fibroblasts in 2007 (Takahashi et al., 2007; Yu et al., 2007). Among many other applications, this exciting discovery opened the door for building a system to investigate the development and function of human brain cells with potential to be reliable, manipulatable, and dynamic. The differentiation of human iPSCs to somatic lineages, including neural cell types, soon followed, and the field moved rapidly to determine the factors and conditions necessary for the specific conversion of iPSCs into neural cell types such as forebrain cortical neurons (Chambers et al., 2009; Shi et al., 2012), midbrain dopaminergic neurons (Cooper et al., 2010), spinal motor neurons (Jiang et al., 2012), GABA interneurons (Liu et al., 2013), astrocytes (Emdad et al., 2012), oligodendrocytes (Wang et al., 2013), and microglia (Abud et al., 2017; Pocock and Piers, 2018). Two dimensional (2D) integrated networks are now produced in a tailored process, adjusted to reflect the cell types required to answer specific questions (Figure 1) that investigate, for example, human brain-specific development (Weick et al., 2011; Ardhanareeswaran et al., 2017) and neuropsychiatric diseases such as Rett Syndrome (Marchetto et al., 2010), schizophrenia (Brennand et al., 2011; Barry et al., 2014; Roussos et al., 2016), and autism (Russo et al., 2019).

Neurodegenerative models, e.g., for Alzheimer's disease and Parkinson's disease (Shi et al., 2017), can now be studied in a species appropriate experimental model, although the issue of a suitable aging and maturation system remains a work in progress. Techniques are being developed to overcome these limitations so as to produce more relevant aging changes at a molecular level. For example, iPSC-derived aging models have been undertaken using direct conversion methods, such as those resulting in induced neurons (iNs) (Vierbuchen et al., 2010) that avoid a return to the pluripotent stem cell stage and in doing so retain, at least partly, age-related epigenetic marks in a variety of iN subtypes (Mertens et al., 2018).

## 3D Models Using Brain Organoids and Scaffolds

One of the major limitations with 2D models is that they cannot recapitulate regional specificity and connectivity as they mature in the normal developing human brain. Although much progress has being made through *in vivo* studies using model animals, human development is unique in many aspects and the brain is perhaps where these particular differences are more noticeable. Studying the human brain in its uniqueness is not only important for gaining insight into organ development, but also crucial for understanding disease and performing preliminary pharmacological tests for new drugs. To begin to overcome the issues caused by such limitations in researching the human brain, scientists have coupled the recent advances in cell reprogramming and previous knowledge of the brain structure to achieve significant progress in the field. Key advances were made by producing three dimensional (3D) networks using non-adherent conditions to generate a 3D cerebral cortex-like structure (Pasca et al., 2015), 3D human multi-cell type culture models using 3D microfluidic platforms (Park et al., 2018), organs-on-a-chip used for high throughput drug screening (Ronaldson-Bouchard and Vunjak-Novakovic, 2018), and a variety of conventional scaffold substrates for 3D cultures (Willerth and Sakiyama-Elbert, 2008). These frameworks improved on 2D networks to include space, time, and inputs to allow the prospects of interrogating new paradigms.

Through the exploration of self-organizing capabilities of the brain, Lancaster et al. in 2013 established an iPSC-derived, 3D culture system that opened up new opportunities for modeling and exploring human brain development. Through self-organization, these cerebral organoids formed specific human cortical brain regions and cell types (Lancaster et al., 2013). For example, the human cortex possesses an expanded outer subventricular zone (OSVZ) that provides the capacity for increased proliferation to augment cortical size and complexity (Hansen et al., 2010), and this is recapitulated in cerebral organoids (Lancaster et al., 2013). In parallel, further progress was achieved by injecting selective molecular signals into the system to induce the formation of specific brain regions (Jo et al., 2016; Qian et al., 2016). Qian et al. used mini bioreactors to produce forebrain organoids with a well-defined OSVZ and demonstrated the presence of outer radial glial cells with selected molecular markers.

Once a basic structure has emerged that resembles a rudimentary human brain and mimics its cellular properties, either by self-organization or via supplementary stimuli, further experiments can be conducted. Molecular characterization can then inform human-specific transcriptomic and epigenomic changes that occur during human brain development (Amiri et al., 2018). Combined with engineering innovations (Brassard and Lutolf, 2019), organoids represent a rich source of possibilities for investigating human brain function and disease, responses to multiple stimuli, and assessing drug treatments.

Although much has been achieved using organoids, a major shortcoming being currently addressed is reproducibility. Even when more sophisticated 3D models of the brains are developed that can accurately recapitulate the complexity and structural features of this human organ, if the models are not reproducible, it can significantly limit their applications. As we are now beginning to understand the processes guiding organoid formation and improving the techniques to measure molecular diversity, future research will focus on minimizing variability. Indeed, advances have been made and results are promising, although studies still suggest there is more to be done to ensure reproducibility across laboratories (Krefft et al., 2018; Velasco et al., 2019).

## 4D Models Connecting 3D Models to Vasculature and Other Body Regions Such as The Gut (Brain/Gut Axis) to Mimic Dynamic Interplay

Although the field is still in its infancy and many advances should occur in the next few years regarding the development of 3D models, researchers have already started to assess its further applications. The next step is to physically connect region-specific organoids to build a more comprehensive functional system. Studies have demonstrated fusion of distinct brain region-derived organoids, such as the medial ganglionic eminence and cortical areas for the analysis of interneuron migration (Xiang et al., 2017), CA1 and CA3 regions in the hippocampus modeling functional connectivity (Sarkar et al., 2018), and dorsal and ventral forebrain (Birey et al., 2017). As these multiple domain structures self-organize in close proximity and form connections, the dynamic interplay can be analyzed as the human brain develops. To further enhance these four dimensional (4D) models, and allow improved growth and maturation, the issues of improving oxygen and nutrient diffusion (McMurtrey, 2016), and incorporating a functional vasculature, have significantly improved recently (Wimmer et al., 2019). Overall, these studies show the rapid advancement and potential in developing a more relevant human brain model that recapitulates functional *in vivo* features.

It may also be possible in the near future to connect brain organoids to other body organ-specific organoids. The brain controls many functions peripherally via the central and peripheral nervous systems and connecting these organs through appropriate neural connectivity could yield valuable inter-organ information. For example, the gut organoids were the first organoids to be developed (Sato et al., 2009) and the brain and gut are connected via enteroendocrine cells that synapse with vagal neurons (Kaelberer et al., 2018). Combining these two organoid systems in the future could allow investigation into the communication between the organs and a better understanding of the underlying principles of food and nutrient sensing and the control of eating and related disorders. The future of organoids could lead to the ability to create almost any system of brain connections, both within the brain as “assembloids” (Pasca, 2019), but also within the whole body wherever the brain is connected via the central and peripheral nervous systems.

## When Does Human Brain Organoid Become Conscious?

As research on brain organoids rapidly progresses, both ethical and philosophical questions arise. As we have illustrated in this review, during the past decade, efforts have culminated in the development of robust and complex neural systems that successfully recapitulate some of the fundamental structures of the brain microscopic and macroscopic environment, self-organize and show spontaneous activity. Remarkably, researchers have recently demonstrated that human cortical-type organoids display electrical patterns that resemble those seen in developing preterm human babies (Trujillo et al., 2019). These organoids showed regular oscillatory activity and synchronous network events during their development that were comparable to those of preterm babies. Spontaneous brain activity in a dish certainly does stir up the consciousness debate and leading organoid researchers have raised concerns and advise caution in order to appropriately utilize this technology (Reardon, 2018). The development of “assembloids” that connect brain regions and peripheral organs, certainly demonstrates the limitless boundaries of this technology through combining engineering, organoid biology and scientific creativity. Furthermore, recent studies demonstrated successful transplantation of cerebral organoids derived from human neural stem cells into mouse brains that showed enhanced integration into the surrounding tissue, and robust differentiation and maturation (Daviaud et al., 2018; Mansour et al., 2018). An unexpected development, however, was the observation of graft-to-host synaptic connectivity, with the growth of human axonal projections extending into the mice brain. The debate is whether these mice are “humanized” and at what point “human” thoughts and behaviors may arise.

The scientific community may need to more clearly define the boundary between biological materials and conscious systems. A general theoretical definition is that the conscious experience is both informative and integrative and, from these axioms, quantitative methods for measuring consciousness can be derived. One objective measure consists of an index resulting from assessing the complexity of the electrical response generated in the remaining of brain in response to local perturbations to the cortex (by magnetic stimulation). This index would only reach higher levels when the response is widespread (which indicates it is integrative) and diversified (which indicates it is informative). If cerebral organoids, or assembloids, begin to shows signs of consciousness, using current clinical standards, the need for forward-thinking recommendations and guidelines will be required.

## Perspective

Human brain development at a molecular level, and the subsequent resulting thought and behavior, have historically been areas of study impossible to be investigated until now. While the technology of stem cell-derived networks and organoids is still somewhat new, the speed of innovation and progress has resulted in the opportunity to learn about the human brain as never before. We are now building human brains for research and can ask questions, for example, regarding what the molecular foundations of behavior and consciousness may be. Or, what are the underpinnings of neurodegenerative and psychiatric disease, how do the brain and peripheral organs communicate, and can we heal and regenerate the brain? These answers may seem distant but, with a relevant human brain model in hand, they are now much closer than ever before.

## Author Contributions

MB and GB conceptualized and wrote the manuscript.

### Conflict of Interest

The authors declare that the research was conducted in the absence of any commercial or financial relationships that could be construed as a potential conflict of interest.
